# Targeting the Cerebellar Circuit: How Exercise Intervention Reshapes White Matter Networks to Alleviate Autism Symptoms

**DOI:** 10.3390/biology15120950

**Published:** 2026-06-18

**Authors:** Kelong Cai, Yifan Shi, Kai Qi, Yufei Liu, Zhimei Liu, Aiguo Chen

**Affiliations:** 1 School of Sport and Brain Health, Nanjing Sport Institute, Nanjing 210014, China; mx120170353@yzu.edu.cn (K.C.); dx120220088@stu.yzu.edu.cn (Y.S.); 2 College of Physical Education, Yangzhou University, Yangzhou 225127, China; 3 Department of Endocrinology, Nanjing Drum Tower Hospital, The Affiliated Hospital of Nanjing University Medical School, Nanjing 210008, China; 4 Department of Physical Education, Gdansk University of Physical Education and Sport, 80-336 Gdansk, Poland; kai.qi@awf.gda.pl (K.Q.); yufei.liu@awf.gda.pl (Y.L.); 5 Department of Rehabilitation Medicine, The Affiliated Hospital of Yangzhou University, Yangzhou 225012, China; dx120200076@yzu.edu.cn

**Keywords:** autism spectrum disorder, exercise intervention, MRI, white matter network, topological properties, randomized controlled trial

## Abstract

Although exercise interventions have been shown to alleviate core symptoms of Autism Spectrum Disorder (ASD), the neural mechanisms underlying these improvements, particularly those involving the White Matter Network (WMN), remain poorly understood. This study investigated the effects of a Mini-Basketball Training Program (MBTP) on core symptoms and WMN in children with ASD. This study adopted a two-site cluster-Randomized Controlled Trial (cRCT) design. Participants from two special education centers in China were randomly assigned to either an intervention group (MBTP) or a control group (CON). The participants underwent a 12-week MBTP. Core symptom assessments and a Diffusion Tensor Imaging (DTI) scan were conducted before and after the intervention. The individual WMNs were constructed using Deterministic Fiber Tracking (DFT). Graph theoretical analysis was applied to examine changes in WMN topological properties after MBTP. The MBTP significantly improved core symptoms in children with ASD, alongside the decreased normalized clustering coefficient (*Gamma*, *γ*), characteristic path length (*Lambda*, *λ*), small-world attributes (*Sigma*, *σ*), and increased global efficiency (*E_glob_*). The nodal clustering coefficient (NCC) increased in the left cuneus (CUN.L) and left cerebellum 9 (CRBL9.L). Notably, the increased NCC in CRBL9.L was significantly correlated with improvements in core symptoms following the MBTP. The improvement in core symptoms in children with ASD following exercise intervention is associated with the remodeling of the WMN, highlighting the cerebellum as a key node in this neural mechanism.

## Simple Summary

Autism Spectrum Disorder affects how children communicate and behave, creating challenges for both children and their families. While exercise is known to help, exactly how it changes the brain is still unclear. This study tested a 12-week Mini-Basketball Training Program for children with autism to see if it could improve their symptoms and reshape connections in their brains. Thirty children took part in the program or continued their usual routines. After the training, children showed clearer improvements in social communication and reductions in repetitive behaviors. Brain scans revealed that the exercise program strengthened connections in important parts of the brain, especially in the cerebellum—a region involved in movement and social skills. These brain changes were directly linked to better behavior. The findings suggest that fun, structured exercise intervention could be a practical, non-medical way to support children with autism, helping them build stronger brain networks and lead more connected lives.

## 1. Introduction

Autism Spectrum Disorder (ASD) is a neurodevelopmental disorder that typically begins to manifest in early childhood and affects social communication and repetitive behaviors throughout the individual’s lifespan [1]. Affecting about 78 million people worldwide, ASD is a condition of global importance on account of both its prevalence and the degree to which it can affect individuals and families [2]. Given the rapidly rising prevalence of autism, various evidence-based interventions, such as applied behavior analysis [3], Discrete Trial Training (DTT) [4], Pivotal Response Training (PRT) [5], and music intervention [6], are widely used in the intervention and treatment of children with ASD. However, the application of these methods relies on a well-established autism rehabilitation system. It is worth noting that most individuals and families worldwide are unable to receive support outside of their own resources [2].

Exercise interventions that can be carried out by guardians at any time and in any place can effectively meet the needs of families worldwide who lack professional medical and health resource support [7]. Numerous previous systematic reviews and meta-analyses have shown that exercise intervention is beneficial for children with ASD in several respects, including physical fitness, motor skills, core symptoms of ASD, attention, and cognitive function [8,9,10,11,12,13]. Despite substantial progress in basic research in this field, the limits of the benefits of exercise intervention for children with ASD are also very clear. These limitations stem from a profound lack of information regarding the neural mechanisms underlying such changes.

To address these gaps in the field, we have conducted extensive preliminary research. Simply put, we have developed a team-based activity, the Mini-Basketball Training Program (MBTP) [14,15], aimed at exploring the underlying neurological mechanisms of exercise interventions in children with ASD. Given the results of a previous study based on multi-modal Magnetic Resonance Imaging (MRI), it has preliminarily been shown that the neural mechanism by which MBTP improves the behavioral symptoms of children with ASD involves changes in brain structure and function in different regions of the brain. For example, evidence based on Diffusion Tensor Imaging (DTI) suggested that increased White Matter Integrity (WMI) was associated with improvements in social communication in children with ASD after MBTP [16]. Another study showed that a reduction in Regional Homogeneity (ReHo) values in the left postcentral gyrus is linked to an enhancement in social communication performance [17]. However, these previous studies are limited, as they focus only on a single brain region. The functioning of the brains of children with ASD depends not only on the activity of a single brain region but also on a widely distributed dynamic system that integrates multiple brain regions, namely, the brain network [18]. Recently, comprehensive evaluations of the brain have emphasized the importance of studying its networks to elucidate the patterns of connectivity changes following exercise interventions in children with ASD. This approach has revealed a reduction in functional connectivity within the Sensorimotor Network (SM) and between the SM and the Salience Network (SN). Additionally, there is a noted decrease in morphological connectivity strength within a cortical–cortical network centered on the left inferior temporal gyrus, as well as within a subcortical–cortical network centered on the left caudate [19]. Most notably, a direct link between the effects of exercise intervention and changes in the White Matter Network (WMN) is yet to be demonstrated in children with ASD. Here, we aim to address this gap in the literature.

In recent years, many studies have investigated white matter connectivity in children with ASD by extracting and integrating DTI image data [20]. Researchers have modeled brain white matter data as a White Matter Network of integrated and separated systems consisting of hundreds of brain regions or nodes and have described its topological properties through graph theoretical methods [21]. Increasing evidence suggests that the development of children with ASD symptoms involves atypical changes in the WMN rather than in a single brain region. For example, a study revealed that changes in WMN Nodal Efficiency (NE) within hubs, including the right middle frontal gyrus, right insula, left median cingulate, and bilateral precuneus, were related to the severity of social communicative impairments in individuals with ASD [22]. This study suggests that the global and nodal topological properties of the brain WMN in ASD are abnormal. Inspired by these observations, our study hypothesizes that the improvement in core symptoms after exercise intervention in children with ASD could be related to changes in WMN topological properties.

To test our hypothesis, we applied a graph theoretical method to reveal the neural mechanisms of the MBTP intervention, focusing primarily on White Matter Networks. We also analyzed the potential association between changes in WMN metrics (global and nodal topological properties) and the core symptoms of children with ASD.

## 2. Materials and Methods

### 2.1. Trial Design, Ethics, Consent, and Registration

The current study was a 12-week, 60-session study employing a cluster-Randomized Trial (cRCT) design with a baseline–post-test approach and a control group. The methodology has been described in detail following the Consolidated Standards of Reporting Trials. This study employed a single-blind design. Participants and their legal guardians were blinded to group allocation. The researcher who generated the randomization sequence using a computer-generated random number table was also blinded to the participants’ identities. However, due to the nature of the exercise intervention, the coaches delivering the Mini-Basketball Training Program could not be blinded to group assignment. Similarly, the outcome assessors who administered the behavioral assessments and analyzed the neuroimaging data were aware of group allocation, although they were not involved in the intervention delivery. The lack of assessor blinding represents a limitation of this study and is discussed further in the Section 4.4.

The authors assert that all procedures contributing to this work comply with the ethical standards of the relevant national and institutional committees on human experimentation and with the Helsinki Declaration. Ethical principles were adhered to throughout the study, and the ethical code (201806001) was obtained from the Ethics Committee of Yangzhou Maternal and Child Health Hospital. This research was registered with the Chinese Clinical Trial Registry (ChiCTR1900024973). Informed consent was obtained from the parents or guardians of all participants.

### 2.2. Participants

Children aged 3 to 6 with ASD, diagnosed by experienced psychiatrists according to DSM-5 criteria, were recruited from two special education centers (Starssailor Education Institution and Chuying Child Development Center) in Yangzhou, China. The following exclusion criteria were used to determine the eligibility of the participants: (1) participation in a structured exercise program within the last 6 months; (2) presence of other psychiatric or neurological disorders; (3) visual or auditory impairments; (4) history of head trauma; (5) medical contraindications to exercise (i.e., acute phase after operation or fracture); (6) current medication with psychoactive drugs; (7) not meeting MRI scan requirements.

**Figure 1:**
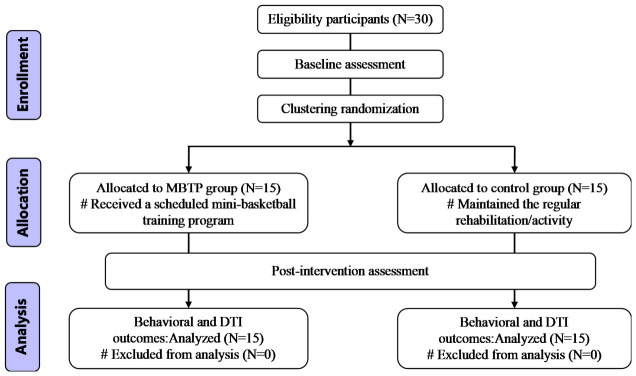
CONSORT flow chart of study participants.

The total sample size was determined using G*power 3.1 software. A repeated measures ANOVA with an effect size of 0.4, a confidence interval of 0.05, and a test efficacy of 0.80 indicated that at least 24 participants were needed for a design involving two groups and two measurement points (pre- and post-tests) [17]. It is important to note that the sample size for this study was determined based on the primary behavioral outcomes. The study was not specifically powered for exploratory neuroimaging analyses. Given the complexity of graph theoretical metrics and the multiple comparisons involved at both global and nodal levels, the current sample size may be underpowered to detect nodal-level effects. Hence, the findings, particularly at the nodal level, should be interpreted with this limitation in mind. Using cluster-based randomization stratified by recruitment site, 30 eligible participants (N = 15 from the Starssailor Education Institution; N = 15 from Chuying Child Development Center) were allocated to either the experimental group (Starssailor) or the treatment-as-usual control group (Chuying). The random allocation of participants was carried out by third-party personnel who did not participate in the experiment. Importantly, to minimize differential expectancy effects, both children with ASD and their parents across the two study sites were blinded to group allocation. Additionally, no opportunities for cross-group social interaction were provided throughout the study period (Figure 1).

### 2.3. Intervention

**Figure 2:**
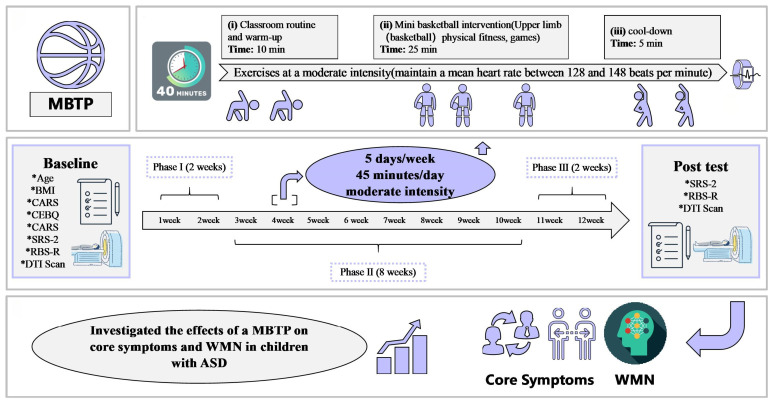
MBTP intervention and procedure.

Details of the Mini-Basketball Training Program (MBTP) intervention protocol (5 days/week, 45 min/day, 12 weeks) were described in our previous study (Figure 2), and no further elaboration is provided here [14,15,16,23].

### 2.4. Core Symptom Measurement

The core symptoms of ASD were evaluated using the Social Responsiveness Scale, Second Edition (SRS-2) (Cronbach’s α = 0.946) [24], which includes five sub-scales, namely, social awareness, social cognition, social communication, social motivation, and autistic mannerisms, for measuring social behavior.

The Repetitive Behavior Scale, Revised Edition (RBS-R) (Cronbach’s α = 0.786) [25], which includes six sub-scales, namely, stereotyped behavior, self-injurious behavior, compulsive behavior, ritualistic behavior, sameness behavior, and restricted behavior, was used to assess repetitive behavior. Higher scores represent more severe core symptoms. The SRS-2 and RBS-R were completed by the parents of participants at baseline and post-test.

### 2.5. DTI Data Acquisition and Preprocessing

Diffusion Tensor Imaging data were acquired using a 3.0T GE Healthcare whole-body high-speed imaging device equipped for echo planar imaging (GE Discovery MR750w 3.0T, Chicago, IL, USA) at the Affiliated Hospital of Yangzhou University. Specific details regarding the scans, the sequence of the DTI scan, and the preprocessing of the DTI data were provided in the previous study [16,26]. Specifically, to ensure the successful completion of the MRI scan for each subject, sedation was administered due to the extended scanning time and the noisy operation of the machine. The sedation management procedure involved instructing the guardian of the autistic child, one day prior to the scan, to keep the participants awake late at night and wake them up early in the morning. Additionally, each subject received an enema containing 10% chloral hydrate at a dose of 0.3 mL/kg (30 mg/kg) every 6 to 8 h, with the maximum dosage not exceeding 10 mL. In cases where the participant did not respond to mild pain stimulation, a medical professional positioned them in a supine position on the scanning bed. Head stabilization was achieved with foam pads, and noises were attenuated via 29 dB-rating earplugs. The same sedation protocol was applied identically across all participants and both time points (baseline and post-test). No adverse events occurred during any of the scanning sessions.

The DTI protocol was as follows: TR = 16,500 ms; TE = 96.2 ms; flip angle = 90°; field of view = 224 × 224 mm^2^; acquisition matrix size = 112 × 112; 70 interleaved slices; voxel size = 2 × 2 × 2 mm^3^; 3 B0 images; 30 diffusion weighted images; and b value = 1000 s/mm^2^. Pipeline for Analyzing Brain Diffusion Images (PANDA), a toolbox in MATLAB R2022b, was used for fully automated processing of diffusion images [27]. The main procedures included preprocessing and producing diffusion metrics in preparation for statistical analysis (local diffusion homogeneity = 7 voxels; normalizing resolution in smooth = 2 mm; and smoothing kernel = 6 mm). The preprocessing steps were executed serially, including converting DICOM files into Nifti images, estimating the brain mask, cropping raw images, correcting for the eddy-current effects, and calculating diffusion tensor metrics. Because participants were young children with ASD, we enforced scan-level quality control beyond the default PANDA pipeline.

To minimize motion artifacts, all participants were scanned under deep sedation, with continuous monitoring by an experienced radiologic technologist. Subject-level motion was summarized using relative translational/rotational changes and framewise displacement derived from the six-parameter motion logs. Datasets would have been rejected if mean framewise displacement exceeded 0.20 mm or if a high proportion of volumes showed excessive relative displacement or artifact signatures. Additionally, we performed visual inspection of PANDA’s quality control snapshots, including raw images, FA maps, brain extraction masks, and registration results, to exclude datasets with marked susceptibility/distortion artifacts, poor skull-stripping leading to registration drift, or widespread invalid tensor solutions. All DTI datasets included in the analysis passed the following quality checks: (1) no apparent motion or susceptibility artifacts on raw images and FA maps; (2) anatomically accurate brain extraction and registration; (3) acceptable tensor fitting residuals. Owing to the sedation scanning protocol, all participants who completed the scan met the quality control criteria, and no dataset was excluded due to scan quality issues.

### 2.6. Construction of White Matter Network

The brain network was described using nodes and edges, which can be defined in various ways. We employed the following methods to define these nodes and edges.

#### 2.6.1. Network Node Definition

First, the space of the DTI was matched with the space of T1 [28]. Therefore, each T1-weighted image is first registered with the B0 image of the DTI through a linear transformation. Then, by applying an affine transformation, the co-registered structural image is mapped to the Montreal Neurological Institute (MNI) T1 template, and a series of nonlinear distortions is used to simulate the affine transformation. The obtained conversion parameters are retrieved and applied to the Automated Anatomical Labeling (AAL) regions, transferring them from the MNI space to the DTI space [29]. Statistical Parametric Mapping (SPM12) (http://www.fil.ion.ucl.ac.uk/spm/) (accessed on 5 May 2025) was applied to conduct the preprocessing, through which nodes have been defined. Previous studies have used the AAL template to divide the brain into 90 regions representing 90 nodes and to construct a structural connectivity network [20,22]. However, the AAL 90 template does not include the cerebellum, which is one of the regulating centers of the body’s basic motor state. It is not only responsible for regulating muscle tone, maintaining body balance, and coordinating fine movements, but it also participates in a wide range of cognitive and emotional functions, including sensory perception, learning, language, and emotional control [30,31,32,33]. Hence, we employed a customized AAL template to divide the cerebral cortex (45 nodes each for the left and right hemispheres) and the cerebellum (26 nodes for the whole cerebellum) into 116 regions. For each subject, the parcellation process was conducted in the DTI native space. This atlas was selected for several reasons. First, it is among the most widely used parcellation schemes in graph theoretical analyses of structural networks in ASD research, facilitating comparability with prior studies. Second, its definition, based on macroscopic anatomical boundaries, ensures high reproducibility across subjects and studies. Third, its moderate granularity (116 regions) balances regional specificity with statistical power, which is particularly relevant given our sample size. Fourth, the AAL atlas is natively supported by the PANDA pipeline, minimizing additional processing variability.

#### 2.6.2. Network Edge Definition

We used the PANDA 1.3.1 toolbox to construct structural networks using Deterministic Fiber Tracking (DFT). Here, the dti_recon and dti_tracker commands of the Diffusion Toolkit (http://trackvis.org/dtk/) (accessed on 7 May 2025) were used to reconstruct all possible fibers within the brain by seeding from all the white matter voxels. The FA threshold was set at FA < 0.2, and the turning angle threshold was set at 45 degrees [34]. For each pair of brain nodes/regions defined above, fibers with two endpoints located within their respective masks were considered to link the two nodes. The edges of the network were then defined based on the number of fiber bundle connections (FN) between regions. If the number of fiber bundles is greater than or equal to 3, it is considered to indicate an effective connection between the two nodes. If it is less than 3, there is either no connection or a pseudo connection between the two nodes. Therefore, FN = 3 is used to establish the edges of the network.

### 2.7. Graph Theoretical Analysis of White Matter Network

All network properties in this study were calculated using the GRETNA toolbox (https://www.nitrc.org/projects/gretna/) (accessed on 7 May 2025) [35]. The global network metrics included clustering coefficient (*C_p_*), normalized clustering coefficient (*Gamma*, *γ*), normalized characteristic path length (*Lambda*, *λ*), small-world attributes (*Sigma*, *σ*), characteristic path length (*L_p_*), global efficiency (*E_glob_*), and local efficiency (*E_loc_*).

At the nodal level, Nodal Clustering Coefficients (NCCs) were calculated for each of the 116 brain regions defined by the AAL 116 template. Considering that the network metrics strongly depend on the network densities, we calculated all network metrics under a wide range of sparsity from 0.05 to 0.5 with a step of 0.05 [19,36]. We computed the Area Under the Curve (AUC) for each network metric across all thresholds to provide a comprehensive measure of the topological organization of brain networks. Statistical comparisons of these nodal metrics were conducted using repeated measures ANOVA models. To manage the issue of multiple comparisons, our strategy was as follows: First, we assessed the overall significance of the main or interaction effects within the ANOVA model for each metric. Bonferroni correction was applied only to the post hoc pairwise comparisons that were conducted following a significant omnibus test. It is important to note that this approach does not constitute a whole-brain, node-level multiple comparison correction across all 116 independent nodal tests. Therefore, the subsequent nodal findings should be treated as exploratory.

### 2.8. Statistical Analysis

All statistical analyses were conducted using Jamovi 2.7 (https://www.jamovi.org/) (accessed on 21 May 2025), with data presented as mean ± standard deviation (M ± SD), and significance set at *p* < 0.05. First, independent sample *t*-tests and chi-square tests were used to assess the homogeneity of demographic data between the MBTP and control groups to control for potential confounding variables. Second, ANOVA analyses with 2 (time: baseline vs. post-test) × 2 (group: MBTP vs. CON) repeated measures were used to investigate the effect of MBTP on the scores of the SRS-2 and RBS-R, as well as the topological properties of the White Matter Networks in children with ASD. Effect sizes for all ANOVA models are reported as partial eta-squared (*η_p_*^2^). If significant interactions were found, further post hoc tests were conducted along with Bonferroni corrections. Finally, Pearson correlation analysis was utilized to explore the relationship between the changes in the topological properties of WMN (post-test minus baseline) and the changes in the SRS-2 and RBS scores (post-test minus baseline) in the MBTP group before and after the exercise intervention.

## 3. Results

### 3.1. Demographic and Clinical Characteristics

The detailed clinical and demographic data for all subjects are shown in Table 1. The chi-square test revealed no statistically significant difference in gender between the two groups of participants (*p* > 0.05). The independent samples *t*-test indicated that there were no statistically significant differences in age, BMI, CARS, CSHQ, CEBQ, and intervention duration (all *p* > 0.05), suggesting homogeneity of demographic and influencing factors between the two groups of participants.

However, considering the potential bias arising from gender imbalance in the sample, the possible influence of age on ASD symptoms and brain development, as well as potential site effects introduced by cluster randomization between the two groups, we incorporated gender, age, and daily duration of routine intervention across different institutions as covariates in subsequent data analyses.

### 3.2. Core Symptoms Results

**Figure 3:**
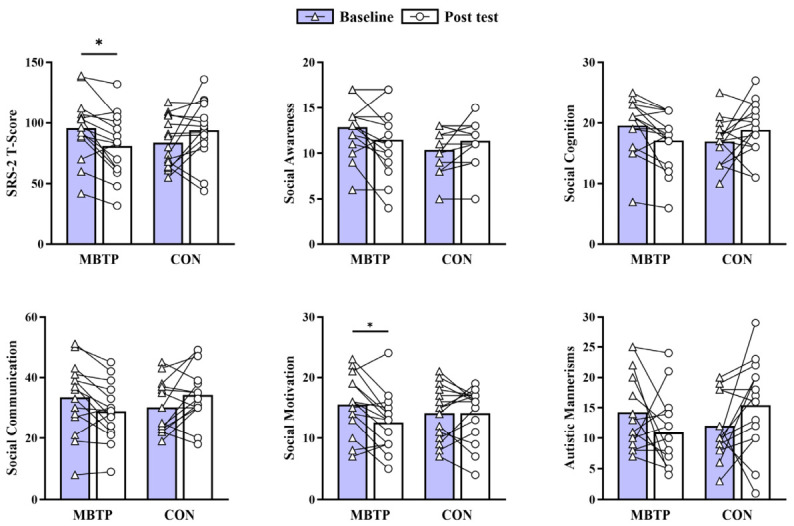
The MBTP effect on SRS-2 performance. Notes: The graph shows the interaction effect between time (baseline, post-test) and group (MBTP, CON) in SRS-2 T-Score, social awareness, social cognition, social communication, social motivation, and autistic mannerisms. * *p* < 0.05 (Bonferroni correction).

In terms of SRS-2, we found a significant group × time interaction on measures for SRS-2 T-score (*p* < 0.01), social awareness (*p* < 0.01), social cognition (*p* < 0.01), social communication (*p* < 0.01), social motivation (*p* < 0.05), and autistic mannerisms (*p* < 0.05) (Table 2). Further post hoc tests with Bonferroni correction revealed that the post-test scores for SRS-2 T-score and social motivation sub-scales were significantly lower than baseline in the MBTP group (all *p* < 0.05), while the CON group did not show significant changes (all *p* > 0.05) (Figure 3), indicating that MBTP significantly improved social dysfunction in children with ASD.

**Figure 4:**
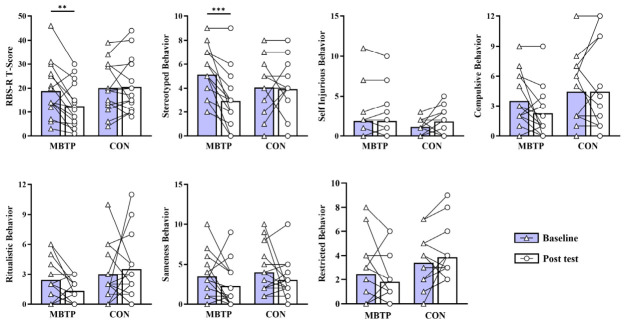
The MBTP effect on RBS-R performance. Notes: The graph shows the interaction effect between time (baseline, post-test) and group (MBTP, CON) in RBS-R T-Score, stereotyped behavior, self-injurious behavior, compulsive behavior, ritualistic behavior, sameness behavior, and restricted behavior. ** *p* < 0.01, *** *p* < 0.001 (Bonferroni correction).

In terms of RBS-R, we found a significant group × time interaction on measures for RBS-R T-score (*p* < 0.05) and stereotyped behaviors (*p* < 0.01) (Table 2). Further post hoc tests with Bonferroni correction indicated that the post-test scores for RBS-R T-score (*p* < 0.05) and stereotyped behavior sub-scales (*p* < 0.001) were significantly lower than baseline in the MBTP group, while the CON group did not show significant changes (all *p* > 0.05) (Figure 4), indicating that MBTP significantly improved repetitive behaviors in children with ASD.

### 3.3. White Matter Network Results

**Figure 5:**
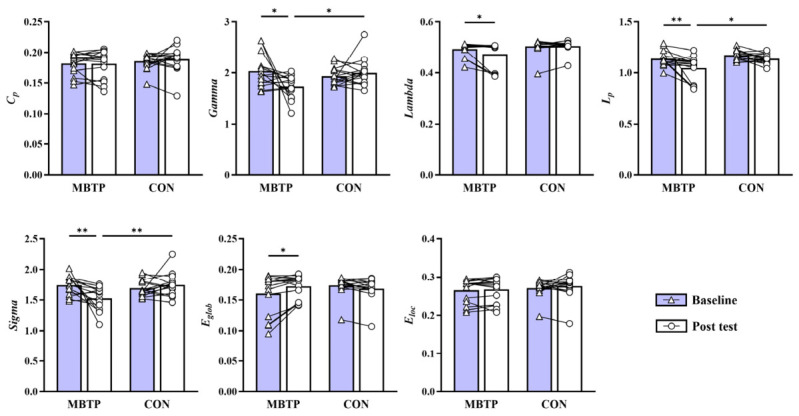
The MBTP effect on global topological properties. Notes: The graph shows the interaction effect between time × group in clustering coefficient (*C_p_*), normalized clustering coefficient (*Gamma*, *γ*), normalized characteristic path length (*Lambda*, *λ*), characteristic path length (*L_p_*), small-world attributes (*Sigma*, *σ*), global efficiency (*E_glob_*), and local efficiency (*E_loc_*). * *p* < 0.05, ** *p* < 0.01 (Bonferroni correction).

In terms of global topological properties, we found a significant group × time interaction on measures for *Gamma* (*p* < 0.05), *Lambda* (*p* < 0.05), *Sigma* (*p* < 0.01), and *E_glob_* (*p* < 0.01) (Table 3). Further post hoc tests with Bonferroni correction revealed that the post-test scores for *Gamma* (*p* < 0.05), *Lambda* (*p* < 0.05), and *Sigma* (*p* < 0.01) were significantly lower than baseline in the MBTP group, while the opposite was true of *E_glob_* (*p* < 0.05), where no significant change was observed in the CON group (all *p* > 0.05). Moreover, the MBTP group measures for *Gamma* (*p* < 0.05) and *Sigma* (*p* < 0.01) were significantly lower than those of the CON group in the post-test, while the baseline did not change (all *p* > 0.05) (Figure 5), indicating that MBTP significantly improved the global topological properties of the WMN in children with ASD.

**Figure 6:**
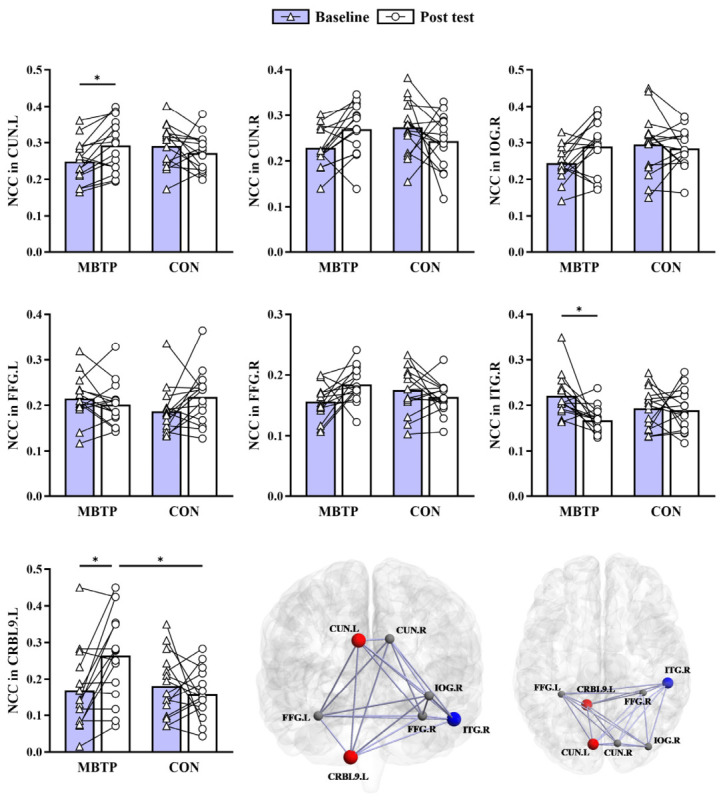
The MBTP effect on nodal topological properties. Notes: The graph shows the significant group × time interactions on measures of the nodal clustering coefficient (NCC) in the left cuneus (CUN.L), right cuneus (CUN.R), right inferior occipital gyrus (IOG.R), left fusiform gyrus (FFG.L), right fusiform gyrus (FFG.R), right inferior temporal gyrus (ITG.R), and left cerebellum 9 (CRBL9.L). * *p* < 0.05 (Bonferroni correction). BrainNet Viewer [37] shows the effect of MBTP in terms of the NCC from baseline to post-test. Red nodes indicate rise, and blue nodes indicate descent.

In terms of nodal topological properties, we found a significant group × time interaction on measures for the NCC of the left cuneus (CUN.L) (*p* < 0.01), the right cuneus (CUN.R) (*p* < 0.05), the right inferior occipital gyrus (IOG.R) (*p* < 0.05), the left fusiform gyrus (FFG.L) (*p* < 0.05), the right fusiform gyrus (FFG.R) (*p* < 0.05), the right inferior temporal gyrus (ITG.R) (*p* < 0.05), and the left cerebellum 9 (CRBL9.L) (*p* < 0.01) (Table 3). Further post hoc tests with Bonferroni correction revealed that the post-test measures for the NCC in CUN.L (*p* < 0.05) and CRBL9.L (*p* < 0.05) were significantly higher than baseline in MBTP participants, while the opposite was true for ITG.R (*p* < 0.05), where no significant change was observed in CON participants (all *p* > 0.05). Moreover, the MBTP group measures for the NCC in CRBL9.L (*p* < 0.05) were significantly higher than those of the CON group in the post-test, while the baseline did not change (*p* > 0.05) (Figure 6), indicating that MBTP significantly improved the nodal topological properties of the WMN in children with ASD.

### 3.4. Correlation Results

**Figure 7:**
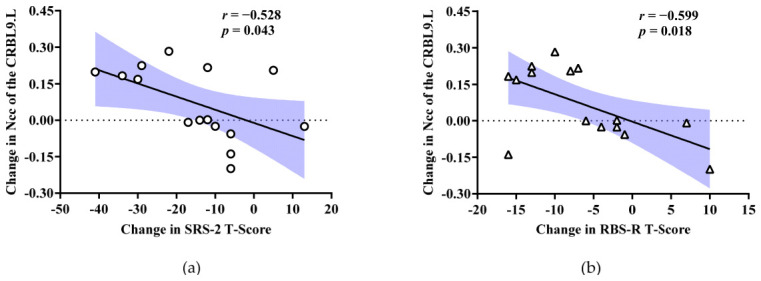
The correlation between change in WMN and change in core symptoms. Notes: Correlation between change in the NCC and change in (**a**) SRS-2 T-Score and (**b**) RBS-R T-Score. Each dot represents the change in a MBTP child (post-test minus baseline). A negative clinical score change value represents an improvement in core symptoms.

Pearson correlation analysis was conducted between the change in WMN topological properties and the change in core symptoms in MBTP participants. The results indicate a significant negative correlation between the NCC of the CRBL9.L region and the SRS-2 T-Score (*r* = −0.528, 95% CI [−0.819, −0.022], *p* = 0.043) and the RBS-R T-Score (*r* = −0.599, 95% CI [−0.851, −0.126], *p* = 0.018) in MBTP participants (Figure 7).

## 4. Discussion

To the best of our knowledge, this study represents the first exploration of the neural mechanisms by which MBTP alleviates core symptoms in children with ASD, specifically examining these effects from the perspective of the WMN. Our findings support our initial hypothesis. Specifically, the 12-week MBTP effectively improved the performance of core symptoms and reshaped the topological properties of the WMN in children with ASD. These results provide direct evidence, at the brain network level, of the neural mechanisms underlying the beneficial effects of exercise interventions on core symptoms in children with ASD. Consequently, this study serves as a significant addition to the existing body of research.

### 4.1. Improved Core Symptoms

This study once again confirms the positive effect of MBTP on social communication and repetitive behaviors in children with ASD, which is consistent with previous research [14,15], particularly regarding the sub-scales of social motivation and stereotyped behavior.

A systematic review and meta-analysis have shown that group-based Organized Physical Activity (OPA) [38], such as Therapeutic Horseback Riding (THR) [39,40], an Outdoor Adventure Program (OAP) [41], and Kata [42,43], can effectively enhance social communication in children with ASD. We suggest that MBTP be considered a typical OPA, including individual and group practice, and providing abundant opportunities for social interactions involving both children and parents. Improvements in the social motivation sub-scales were observed after the MBTP. A prior study involving a 12-week THR intervention found a significant increase in social motivation in individuals with ASD [44]. The social motivation of children with ASD can be maximized through multi-role social interactions, such as those involving children, peers, teachers, and parents.

According to the theoretical operant nature of stereotypy [45], repetitive behaviors are learned and maintained via their sensory consequences, satisfying internal sensory needs in children with ASD. Therefore, by providing equivalent but alternative sensory stimulation, stereotypic behavior can be reduced [46,47]. In one study, hand-flapping stereotypy was significantly reduced, but body-rocking stereotypy following the ball-tapping exercise intervention was not [48]. The MBTP includes a wide range of basketball skills and motor learning content, requiring children to engage their sensory perception and motor functions. In this process, the external stimuli that children receive are similar to those generated by stereotyped behaviors, which helps reduce the occurrence of repetitive behaviors.

### 4.2. Decreased Small-World Attributes and Increased Global Efficiency

Our results find that following MBTP, children with ASD had significantly decreased *Gamma*, *Lambda*, *Sigma*, and *L_p_* and increased *E_glob_* in global topological properties.

The small-world model is well suited for complex brain dynamics because it integrates and distributes information processing to maximize the efficiency of information dissemination at a relatively low cost [36]. We found that both the MBTP group and the control group exhibited topological small-world network properties (*Sigma* > 1), and the small-world properties were significantly decreased after MBTP. Interpreting this finding requires the integration of existing literature and appropriate caution. Our interpretive framework is informed by several prior studies which have reported that individuals with ASD, including children in comparable age groups, can exhibit elevated small-world properties in functional or structural brain networks compared with Typically Developing (TD) controls, often interpreted as reflecting “over-differentiation” or altered global integration efficiency [20]. Consequently, the post-intervention reduction in these parameters may suggest a shift in network topology towards a more typical developmental profile. It is crucial to emphasize, however, that this “normalization” interpretation remains speculative in the absence of a direct comparison with a matched TD control group in our study.

In addition, *E_glob_* denotes the measurement of the information transmission capacity between far-apart brain regions, which is mainly related to long-distance connections. An abnormal *E_glob_* in the WMN topology of individuals with ASD has been reported, but the findings are frequently inconsistent. One study involving preschool children with ASD showed that the ASD group exhibited a significantly increased *E_glob_* in their WMN. This suggests that the white matter structures of children with ASD aged 2–6 are overconnected [49]. However, another study on MZCo-ASD noted decreased *E_glob_* [20], and a study of ASD in infants also found significantly decreased *E_glob_* across the temporal, parietal, and occipital lobes of high-risk infants classified as having ASD [50], which aligns with our results. We found that the *E_glob_* significantly increased after MBTP intervention in the MBTP group. These changes imply that MBTP improves the information transmission capacity between far-apart brain regions. Specifically, distributed cortical–subcortical connections undergo plastic changes in service to a more efficient synergy between the brain regions that automatically transmit and integrate local information.

### 4.3. Decreased Nodal Clustering Coefficient of ITG.R and Increased Nodal Clustering Coefficient of CUN.L and CRBL9.L

The clustering coefficient of nodes selected in this study describes the closeness between these nodes and other nodes in the WMN, reflecting the functional differentiation mechanism of the cerebral cortex, specifically the proximity of local brain functional neurons to other neurons. Our results indicate that children with ASD exhibit a decreased nodal clustering coefficient of ITG.R and an increased nodal clustering coefficient of CUN.L and CRBL9.L after MBTP.

First, we demonstrated a decreased nodal clustering coefficient of the ITG after 12 weeks of MBTP. In agreement with our study, Zhou found a decrease in the Nodal Efficiency (NE) and Nodal Degree (DC) of the ITG in children with ASD after MBTP [19]. The ITG is involved in visual perception and processing, which relates to the transmission of information back and forth, and it plays a key role in language and emotional cognition [51]. Evidence from preschool boys with High-Functioning Autism (HFA) shows a significant negative correlation between the repetitive behavior score and increased ITG [52]. Another study showed that there was increased Functional Connectivity (FC) between the right medial orbital frontal cortex and the bilateral Inferior Temporal Gyrus (ITG) in subjects with ASD; however, there was no correlation between the abnormal FC values and clinical scales [53]. More importantly, given the significance of the abnormal structure of the ITG in ASD patients, our results show that a decreased nodal clustering coefficient of the ITG may partly explain the improvement in core symptoms after MBTP.

Second, in our study, the increased nodal clustering coefficient of CUN.L was found in children with ASD after MBTP. The cuneus/precuneus plays a pivotal role within the frontoparietal network by serving as a bridge between the parietal and prefrontal regions [21]. It has been reported that the middle temporal gyrus and other temporal areas have lower effective connectivity to the CUN in individuals with autism, and these were correlated with the ADOS scores [54]. Moreover, a study reported significantly greater activation in the left CUN in children with ASD. Further results indicate that those with higher AQ scores show greater activity in the left CUN [55]. Therefore, given the significance of abnormal CUN function in children with ASD, our findings, which indicate an increased nodal clustering coefficient in CUN, may also partially elucidate the improvement in core symptoms observed after MBTP.

Finally, we find that the node clustering coefficient in CRBL9.L increased significantly after MBTP. More importantly, correlation analysis further revealed that the increased NCC in CRBL9.L was negatively associated with the improved performance of core symptoms in children with ASD. The cerebellum, traditionally associated with motor control, has increasingly been recognized for its role in cognitive and emotional processes, which are often impaired in individuals with ASD. This recognition has led to a growing body of research investigating the contribution of cerebellar dysfunction to ASD symptomatology. A critical examination of the literature reveals several mechanisms through which developmental cerebellar dysfunction may contribute to ASD, including structural abnormalities, impaired connectivity, and altered neurotransmission. Structural abnormalities in the cerebellum have been consistently observed in individuals with ASD. Studies utilizing advanced imaging techniques have identified reduced gray matter in specific cerebellar lobules, such as Crus I/II, which correlate with the severity of core ASD symptoms, including social interaction deficits and repetitive behaviors [56]. These findings are supported by research indicating that children with ASD exhibit reduced cerebellar vermal volumes, particularly in lobules VI–VII, which are associated with cognitive and emotional processing [57]. Moreover, abnormal cerebellar development, characterized by Purkinje cell loss and cerebellar hypoplasia, has been linked to disruptions in glutamatergic projections to the prefrontal cortex, potentially affecting dopamine release and contributing to the cognitive and behavioral symptoms of ASD [58]. In addition to structural changes, altered connectivity between the cerebellum and other brain regions has been implicated in ASD. Studies have demonstrated atypical cerebrocerebellar connectivity, with increased functional connectivity correlating with executive function impairments in ASD individuals [59]. This altered connectivity may reflect a broader disruption in the coordination between the cerebellum and neocortex, as evidenced by multimodal imaging studies showing atypical structural and functional coupling in ASD [60]. These connectivity issues are further compounded by microstructural abnormalities in cerebellar pathways, such as the superior cerebellar peduncle, which have been associated with motor dysfunction in children with ASD [61]. These studies suggest that abnormalities in the cerebellum may play critical roles in the neurobiology of ASD. To our knowledge, our study is among the first to construct a WMN using an AAL 116 template that includes the cerebellum as a network node. We found that the increased node clustering coefficient of CRBL9.L after MBTP was related to improvements in core symptoms and drove brain changes towards a more normal-like neuroanatomy. This is an exciting find because this is the first study to validate the remodeling effect of exercise intervention on the autistic cerebellum at the level of the WMN. However, while the present findings indicate an association between White Matter Network remodeling and clinical improvement, they do not permit causal inference. Future studies incorporating longitudinal mediation frameworks or randomized controlled designs will be required to determine whether the observed network changes contribute causally to symptom gains or instead co-occur as related but mechanistically distinct treatment-linked adaptations.

### 4.4. Limitations

First, the intervention was implemented using a two-cluster design, with each study arm located in a separate institution. Although we accounted for multiple individual- and institution-level covariates, residual confounding due to unmeasured institutional differences (e.g., in clinical practices or resources) cannot be ruled out. Therefore, the observed effects may reflect either the intervention itself or underlying variations between sites. Future multi-center cluster-randomized trials, with institutions as the unit of randomization, are needed to better isolate the true intervention effect.

Second, although the sample size provided adequate power for the primary behavioral outcomes, it may be underpowered for exploratory neuroimaging analyses, particularly for the graph theoretical network metrics involving multiple global and nodal measures. This increases the risk of false positives and limits our ability to detect small-to-moderate effects. Although we employed multiple comparison correction, nodal-level findings should be interpreted as preliminary and require replication in larger, independently sampled cohorts.

Lastly, this study adopted a single-blind design in which participants and their guardians were blinded to group allocation, but the outcome assessors were not. The lack of assessor blinding introduces a potential source of bias, particularly for behavioral assessments that involve subjective judgment. Although the primary neuroimaging outcomes are objective and less susceptible to rater expectation, the possibility of unintended bias cannot be entirely excluded. Future studies should consider implementing double-blind designs to further minimize assessment bias.

## 5. Conclusions

In conclusion, our research provides further evidence supporting the efficacy of the MBTP intervention in alleviating the core symptoms of children with ASD. Using the graph theoretical method in an exploratory analysis, we observed preliminary evidence that MBTP may be associated with alterations in the topological properties of the WMN. Notably, changes in the nodal clustering coefficient within the CRBL9.L region were preliminarily linked to improvements in core symptoms, suggesting a potential shift in brain neuroanatomy toward a more typical developmental trajectory. These exploratory findings indicate that the neural mechanism underlying exercise-induced changes in core symptoms may involve remodeling of the WMN. However, these nodal-level results require replication in larger samples to confirm their specificity and reliability.

## Figures and Tables

**Table 1:** Demographic characteristics (M ± SD).

Variable	MBTP (*N* = 15)	CON (*N* = 15)	*t/χ* ^2^	*p*
Gender (male/female)	12/3	14/1	1.153	0.283
Age (year)	5.133 ± 0.611	4.900 ± 1.105	0.7151	0.480
BMI (kg/m^2^)	15.648 ± 1.170	16.466 ± 2.204	−1.270	0.215
CSHQ	56.800 ± 5.060	57.733 ± 12.115	−0.275	0.785
CEBQ	55.800 ± 7.674	52.400 ± 20.202	0.501	0.620
CARS	41.200 ± 7.233	39.200 ± 3.986	0.938	0.356
Intervention Duration (h/day)	4.467 ± 0.990	4.800 ± 0.862	−0.983	0.334

Notes: CSHQ = Children’s Sleep Habits Questionnaire; CEBQ = Child Eating Behavior Questionnaire; CARS = Childhood Autism Rating Scale.

**Table 2:** Core symptom results (M ± SD).

Variables	MBTP (N = 15)		CON (N = 15)		*F*	*p*	*η_p_* ^2^
	Baseline	Post-Test	Baseline	Post-Test			
The Social Responsiveness Scale, Second Edition (SRS-2)							
SRS-2 T-score	95.667 ± 25.612	80.933 ± 25.988	83.533 ± 20.357	94.000 ± 24.562	13.482	0.001	0.325
Social Awareness	12.866 ± 3.067	11.466 ± 3.888	10.333 ± 2.410	11.333 ± 2.581	8.920	0.006	0.241
Social Cognition	19.533 ± 4.673	17.066 ± 4.697	16.933 ± 3.712	18.866 ± 4.437	9.226	0.005	0.248
Social Communication	33.466 ± 11.667	28.866 ± 9.470	30.133 ± 8.348	34.266 ± 9.207	11.545	0.002	0.292
Social Motivation	15.533 ± 4.808	12.600 ± 4.461	14.133 ± 4.470	14.133 ± 4.421	5.569	0.029	0.158
Autistic Mannerisms	14.266 ± 6.158	10.933 ± 5.957	12.000 ± 5.264	15.400 ± 7.327	5.924	0.022	0.175
The Repetitive Behavior Scale, Revised Edition (RBS-R)							
RBS-R T-score	18.867 ± 11.344	12.467 ± 9.425	20.067 ± 10.229	20.600 ± 11.160	6.499	0.017	0.188
Stereotyped Behavior	5.133 ± 2.099	2.933 ± 2.374	4.066 ± 2.576	3.933 ± 2.631	10.413	0.003	0.271
Self-Injurious Behavior	1.866 ± 3.159	1.866 ± 3.020	1.133 ± 0.990	1.800 ± 1.612	2.154	0.153	0.071
Compulsive Behavior	3.533 ± 2.503	2.266 ± 2.404	4.466 ± 3.461	4.466 ± 4.340	1.883	0.181	0.063
Ritualistic Behavior	2.400 ± 2.164	1.333 ± 1.175	3.000 ± 2.699	3.533 ± 3.204	2.078	0.160	0.069
Sameness Behavior	3.466 ± 2.722	2.266 ± 2.658	4.000 ± 3.000	3.000 ± 2.390	0.030	0.863	0.001
Restricted Behavior	2.466 ± 2.587	1.800 ± 2.166	3.400 ± 2.063	3.866 ± 2.166	2.837	0.103	0.092

Notes: The table shows the interaction effect between time and group in SRS-2 and RBS-R performance. *η_p_*^2^ = partial eta squared.

**Table 3:** WMN topological properties results (M ± SD).

Variables	MBTP (N = 15)		CON (N = 15)		*F*	*p*	*η_p_* ^2^
	Baseline	Post-Test	Baseline	Post-Test			
Global Topological Properties							
*C_p_*	0.182 ± 0.018	0.181 ± 0.023	0.185 ± 0.013	0.189 ± 0.021	0.496	0.487	0.017
*Gamma*	2.029 ± 0.321	1.731 ± 0.230	1.927 ± 0.175	1.996 ± 0.262	6.141	0.020	0.124
*Lambda*	0.494 ± 0.027	0.474 ± 0.052	0.505 ± 0.032	0.505 ± 0.022	5.194	0.031	0.023
*L_p_*	1.136 ± 0.076	1.045 ± 0.124	1.174 ± 0.050	1.137 ± 0.049	2.030	0.165	0.067
*Sigma*	1.742 ± 0.157	1.523 ± 0.191	1.691 ± 0.135	1.746 ± 0.187	9.947	0.004	0.262
*E_glob_*	0.161 ± 0.033	0.172 ± 0.019	0.174 ± 0.017	0.169 ± 0.019	12.538	0.001	0.309
*E_loc_*	0.265 ± 0.031	0.267 ± 0.033	0.276 ± 0.031	0.271 ± 0.023	0.205	0.654	0.007
Nodal Topological Properties							
CUN.L	0.248 ± 0.057	0.292 ± 0.071	0.291 ± 0.061	0.272 ± 0.051	8.677	0.006	0.237
CUN.R	0.229 ± 0.044	0.270 ± 0.057	0.274 ± 0.061	0.244 ± 0.060	7.093	0.013	0.202
IOG.R	0.244 ± 0.048	0.289 ± 0.074	0.295 ± 0.085	0.284 ± 0.053	4.786	0.037	0.146
FFG.L	0.215 ± 0.050	0.202 ± 0.048	0.187 ± 0.051	0.218 ± 0.059	4.256	0.049	0.132
FFG.R	0.156 ± 0.030	0.185 ± 0.029	0.175 ± 0.039	0.163 ± 0.027	6.793	0.015	0.195
ITG.R	0.221 ± 0.046	0.168 ± 0.030	0.194 ± 0.044	0.190 ± 0.045	4.526	0.042	0.139
CRBL9.L	0.170 ± 0.108	0.265 ± 0.123	0.181 ± 0.087	0.158 ± 0.068	8.716	0.007	0.236

Notes: The table shows the interaction effect between time and group in global and nodal topological properties. *η_p_*^2^ *=* partial eta squared. CUN = cuneus; IOG = inferior occipital gyrus; FFG = fusiform gyrus; ITG = inferior temporal gyrus; CRBL9 = cerebellum 9; *C_p_* = clustering coefficient; *L_p_* = characteristic path length; *E_glob_* = global efficiency; *E_loc_* = local efficiency.

## Data Availability

The raw data supporting the conclusions of this article will be made available by the authors on request.

## Abbreviations

The following abbreviations are used in this manuscript:

*γ*	Normalized clustering coefficient
*λ*	Characteristic path length
*σ*	Small-world attributes
AAL	Automated anatomical labeling
ABA	Applied behavior analysis
ASD	Autism spectrum disorder
AUC	Area under the curve
BMI	Body mass index
CARS	Childhood autism rating scale
CEBQ	Child eating behavior questionnaire
CON	Control group
*C_p_*	Clustering coefficient
CRBL9.L	Left cerebellum 9
cRCT	Cluster-randomized controlled trial
CSHQ	Children’s Sleep Habits Questionnaire
CUN.L	Left cuneus
CUN.R	Right cuneus
Dc	Nodal degree
DFT	Deterministic fiber tracking
DSM-5	Diagnostic and statistical manual of mental disorders, fifth edition
DTI	Diffusion tensor imaging
DTT	Discrete trial training
*E_glob_*	Increased global efficiency
*E_loc_*	Local efficiency
FC	Functional connectivity
FFG.L	Left fusiform gyrus
FFG.R	Right fusiform gyrus
HFA	High-functioning autism
IOG.R	Right inferior occipital gyrus
ITG.R	Right inferior temporal gyrus
*L_p_*	Characteristic path length
MBTP	Mini-basketball training program
MNI	Montreal neurological institute
MRI	Magnetic resonance imaging
MZCo-ASD	Monozygotic twins with ASD
NCC	Nodal clustering coefficient
Ne	Nodal efficiency
OPA	Organized physical activity
PANDA	Pipeline for analyzing brain diffusion images
PRT	Pivotal response training
RBS-R	Repetitive behavior scale, revised edition
SM	Sensorimotor network
SN	Salience network
SPM12	Statistical parametric mapping
SRS-2	Social responsiveness scale, second edition
TD	Typical development
THR	Therapeutic horseback riding
WMN	White matter network
